# Sprouted Fenugreek-Enriched Wheat Flatbread Attenuates Postprandial Glycemic and Insulinemic Responses in Healthy Adults: A Randomized Crossover Pilot Study

**DOI:** 10.3390/nu18172811

**Published:** 2026-08-27

**Authors:** Sumaira Saeed, Tariq Ismail, Ahmad Mujtaba Noman, Muhammad Ibrahim, Maria Liccardo, Paola Stiuso

**Affiliations:** 1 Department of Precision Medicine, University of Campania “Luigi Vanvitelli”, 80138 Naples, Italy; sumaira.saeed@unicampania.it (S.S.); muhammad.ibrahim@unicampania.it (M.I.); maria.liccardo@unicampania.it (M.L.); 2 Department of Food Science & Technology, Faculty of Food Science & Nutrition, Bahauddin Zakariya University, Multan 60800, Pakistan; 3 Department of Human Nutrition, Faculty of Food Science and Nutrition, Bahauddin Zakariya University, Multan 60800, Pakistan; ahmadmujtaba493@gmail.com; 4 Unit of Dietetics and Sports Medicine, University of Campania “Luigi Vanvitelli”, 80138 Naples, Italy

**Keywords:** sprouted fenugreek, insulinemic response, wheat flatbread, antioxidant activity, functional foods

## Abstract

**Background/Objectives:** The development of functional staple foods with improved metabolic properties represents a practical dietary strategy for the prevention of type 2 diabetes. This study investigated the effects of incorporating sprouted fenugreek (*Trigonella foenum-graecum* L.) into wheat-based flatbread (chapatti) on postprandial glycemic and insulinemic responses in healthy adults. **Methodology:** Wheat (cv. Galaxy 2013) and fenugreek seeds were germinated for up to 96 h and analyzed for nutritional composition and antioxidant activity. Composite flours containing 0%, 2.5%, 5%, and 7.5% sprouted fenugreek were formulated and evaluated for sensory acceptability. Eighteen healthy participants (18–25 years; BMI 21–22.5 kg/m^2^) consumed chapattis containing 7.5% sprouted fenugreek or white flour as a control in a randomized crossover design. Blood glucose and serum insulin were measured at 0, 30, 60, and 120 min postprandially. **Results:** Sprouting significantly increased protein, dietary fiber, and antioxidant capacity in both wheat and fenugreek (*p* < 0.001). Total phenolic content in wheat increased from 55.82 to 110.17 mg GAE/100 g after 96 h of germination, accompanied by a significant rise in DPPH radical scavenging activity. The formulation containing 7.5% sprouted fenugreek showed the highest sensory acceptability (7.52/9). Fenugreek-supplemented chapattis significantly reduced postprandial glucose and insulin responses (*p* < 0.05) compared with control. **Conclusion:** Incorporation of 7.5% sprouted fenugreek into wheat flatbread improves nutritional quality while attenuating glycemic and insulinemic responses, suggesting a practical dietary strategy for metabolic health and diabetes prevention.

## 1. Introduction

Metabolic disorders including obesity, dyslipidemia, and metabolic syndrome collectively may contribute to creating a strong predisposition to type 2 diabetes mellitus (T2DM) by promoting chronic insulin resistance and impaired glucose homeostasis. T2DM represents one of the most pressing global health challenges and is projected to affect 642 million people worldwide by 2040 [1]. The disease also imposes a substantial economic burden, with global healthcare costs estimated at USD 1.31 trillion annually. Furthermore, diabetes-related complications, including cardiovascular disease, nephropathy, retinopathy, and neuropathy, contribute significantly to increased morbidity and mortality globally [2]. Dietary interventions aimed at moderating postprandial glycemic excursions are widely recognized as essential components of diabetes prevention and management strategies [3]. Low-glycemic-index (GI) foods produce slower and more sustained increases in blood glucose concentrations, thereby improving glycemic control and reducing metabolic stress while lowering the risk of diabetes-related complications. Given the widespread consumption of cereal-based staples, reformulation of commonly consumed products represents a sustainable and culturally adaptable approach to metabolic risk reduction [4].

Wheat (*Triticum aestivum*) is a primary staple food for approximately 40% of the global population, providing essential carbohydrates, proteins, dietary fiber, vitamins, and minerals. In South Asian countries, wheat-based flatbreads such as chapatti are consumed daily by millions of people. However, refined wheat flour products typically exhibit high GI values (70–75), which can lead to pronounced postprandial hyperglycemia [5]. Improving nutritional quality and reducing the glycemic impact of wheat-based foods could therefore yield substantial public health benefits.

Fenugreek (*Trigonella foenum-graecum* L.) is an annual leguminous plant widely cultivated in India, Mediterranean regions, and North Africa and has been traditionally valued for its nutritional and medicinal properties. Fenugreek seeds contain approximately 45–50% dietary fiber (primarily galactomannan), 20–25% protein, and a wide range of bioactive compounds, including essential amino acids such as 4-hydroxyisoleucine, flavonoids, saponins, and alkaloids [6]. Numerous studies have demonstrated the hypoglycemic, hypolipidemic, and antioxidant activities of fenugreek, mediated through mechanisms such as delayed gastric emptying, reduced intestinal glucose absorption, enhanced insulin sensitivity, and increased pancreatic insulin secretion [6]. Nevertheless, the bitter taste and characteristic aroma of fenugreek limit its incorporation into commonly consumed foods. Recent evidence has further highlighted the growing interest in fenugreek as a functional food ingredient owing to its rich content of bioactive compounds, including polyphenols, flavonoids, saponins, alkaloids, and soluble fibers, which collectively contribute to its antioxidant, anti-inflammatory, and glucose-lowering properties. These characteristics make fenugreek an attractive candidate for the development of functional cereal-based foods aimed at improving metabolic health [7].

Sprouting (germination) is a cost-effective bioprocessing technique that enhances the nutritional and functional properties of grains and seeds through enzymatic and biochemical modifications. During germination, complex macromolecules are hydrolyzed into more bioavailable forms, anti-nutritional factors are reduced, and bioactive compounds such as vitamins, phenolics, and antioxidants are synthesized or released. In wheat, sprouting has been reported to improve protein digestibility, increase lysine content, decrease phytic acid, and enhance mineral bioavailability [8]. Similarly, sprouted fenugreek exhibits improved protein content, reduced bitterness, enhanced antioxidant activity, and superior sensory attributes compared with raw seeds.

The integration of sprouted wheat and fenugreek into functional staple foods represents a promising strategy for the development of nutritionally enriched, diabetes-friendly products. However, there is limited systematic evidence regarding the impact of sprouted fenugreek incorporation into wheat-based foods on postprandial glycemic and insulinemic responses in humans. Moreover, optimal inclusion levels that balance nutritional improvement, metabolic benefits, and sensory acceptability remain to be established [9]. Therefore, the present study aimed to evaluate the effects of sprouting duration on the nutritional composition and antioxidant properties of wheat and fenugreek, and to develop and optimize wheat–fenugreek composite flours for chapatti production. In addition, the sensory acceptability of chapattis enriched with sprouted fenugreek was assessed, and the postprandial glycemic and insulinemic responses to these products were determined in healthy, normoglycemic adults. We hypothesized that the incorporation of sprouted fenugreek would enhance the nutritional quality of the product while significantly attenuating postprandial glycemic and insulinemic responses compared with conventional wheat chapattis.

## 2. Materials and Methods

### 2.1. Raw Materials Procurement

Wheat grains (*Triticum aestivum* L., cultivar Galaxy 2013) and fenugreek (*Trigonella foenum-graecum* L.) seeds were procured from a local certified supplier in Multan, Pakistan. The plant materials were visually inspected to ensure uniform size, maturity, and the absence of physical damage, insect infestation, or fungal contamination. Grains and seeds were manually sorted to remove foreign materials and impurities, thoroughly washed with distilled water, and air-dried before being subjected to the germination procedure. Analytical-grade chemicals and reagents were purchased from Sigma-Aldrich (St. Louis, MO, USA) and Merck (Darmstadt, Germany).

### 2.2. Sprouting Procedure

Wheat and fenugreek were germinated separately using a standardized soak–drain method. Seeds were soaked in distilled water (24 h, 25 ± 2 °C) [10] drained, and incubated in a germination chamber at 90–95% relative humidity for 24–96 h with periodic rinsing. Germinated samples were dried in a forced-air hot-air oven Memmert UF55 (Memmert GmbH, Schwabach, Germany) at 70 °C until constant weight was achieved. The dried samples were then milled using a 60-mesh sieve and stored in airtight containers at room temperature until analysis [11,12].

### 2.3. Composite Flour Formulation and Chapatti Preparation

Composite flours were prepared by incorporating 0%, 2.5%, 5%, and 7.5% sprouted flour (SF) into sprouted wheat flour (SW). Chapattis were prepared following standardized procedures. Each composite flour was mixed with water to form soft dough, rested for 30 min, and divided into 77.5 g portions [13]. Dough was rolled into flat circles (18 cm diameter, 3 mm thickness) and baked on a preheated griddle at 210 °C for 1–2 min per side. Fresh chapattis were prepared on the day of testing.

### 2.4. Nutritional and Antioxidant Analysis

Moisture, protein, fat, fiber, ash, and carbohydrate contents were determined using AOAC official methods (17th edition). Moisture was determined by oven-drying at 100 °C (Method 925.09); ash content by muffle furnace incineration at 550 °C (Method 923.03); protein by the Kjeldahl method (Method 979.09; conversion factor 6.25); fat by Soxhlet extraction (Method 920.39); fiber by acid–alkali digestion (Method 962.09); and carbohydrates by the phenol–sulfuric acid method using glucose as the standard [14].

Total Phenolic Content (TPC): Total phenolic content was determined using the Folin–Ciocalteu method. Flour extracts (50 mg in 25 mL of acetone:methanol:water:formic acid, 40:40:20:0.1, *v*/*v*/*v*/*v*) were incubated at 60 °C and subsequently filtered. Aliquots (1 mL) were mixed with Folin–Ciocalteu reagent and 7.5% sodium carbonate solution and incubated for 2 h at room temperature. Absorbance was measured at 726 nm using a UV-1800 UV–Vis spectrophotometer (Shimadzu Corporation, Kyoto, Japan). Results were expressed as mg gallic acid equivalents (GAE) per 100 g dry weight [15].

DPPH Radical Scavenging Activity: Samples (50–100 μL) were mixed with 3 mL of 0.1 mM DPPH solution in methanol and incubated in the dark for 30 min. Absorbance was measured at 517 nm using the same UV-1800 UV–Vis spectrophotometer (Shimadzu Corporation, Kyoto, Japan). Antioxidant activity was expressed as percentage radical scavenging activity, using ascorbic acid as the reference standard [16].

### 2.5. Sensory Evaluation of Products

Sensory quality was rated by 24 trained panelists using a 9-point hedonic scale (1 = dislike extremely, 9 = like extremely). Panelists assessed freshly prepared chapattis for appearance, color, texture, taste, chewing ability, folding ability, odor, and overall acceptability. All samples were coded and served randomly under controlled conditions (bright light, neutral odor, minimal noise). Palates were cleansed with water between samples.

The initial sensory evaluation using sprouted wheat fenugreek-based formulations was discontinued due to rejection by panelists due to bitter taste, dark color and crisp texture. Therefore, only reformulated samples were evaluated. The treatment concentrations were the same but sprouted wheat flour was replaced by plain wheat flour.

### 2.6. Instrumental Colour Analysis

Colour of chapattis prepared with different levels of sprouted and unsprouted fenugreek was evaluated using the CIE Lab* colour system. Freshly baked chapattis were cooled to room temperature before colour measurements were performed on the upper surface at three randomly selected points per sample using a Chroma Meter CR-400 (Konica Minolta, Osaka, Japan) calibrated against a standard white calibration tile before use. The instrument provided the colour coordinates L* (lightness), a* (red–green), and b* (yellow–blue). Mean ± standard deviation (SD) values were calculated for each formulation (0, 2.5, 5.0, and 7.5 g fenugreek; sprouted and unsprouted).

### 2.7. Glycemic and Insulinemic Response Testing

#### 2.7.1. Study Participants

The study was conducted at the Faculty of Food Science and Nutrition, Bahauddin Zakariya University, Multan, Pakistan. Participants were recruited among university students at Bahauddin Zakariya University. Eligible students were screened according to the predefined inclusion and exclusion criteria before enrollment in the study. Eighteen healthy normoglycemic volunteers (9 males and 9 females), aged 18–25 years, with a body mass index (BMI) between 21.0 and 22.5 kg/m^2^ and fasting blood glucose levels < 6.0 mmol/L, were enrolled. Participants were screened for metabolic, gastrointestinal, and other chronic diseases and were not taking medications known to affect glucose metabolism. The study was conducted in accordance with the Declaration of Helsinki and approved by the Institutional Ethics Committee (Approval No. EC134/2024). Written informed consent was obtained from all participants prior to enrollment.

#### 2.7.2. Study Design

A randomized crossover design was employed. Each participant consumed three test meals on separate occasions in a randomized order and served as his or her own control. A washout period of at least three days was implemented between test sessions to minimize potential carry-over effects and to ensure that postprandial metabolic responses returned to baseline before the subsequent intervention.

The test meals consisted of:

- Control wheat chapatti (0% fenugreek);
- Chapatti supplemented with 7.5% sprouted fenugreek flour;
- Chapatti supplemented with 7.5% unsprouted fenugreek flour.

All test meals provided 50 g of available carbohydrates. Participants were instructed to fast overnight (10–12 h) before each test session and to avoid alcohol consumption, strenuous physical activity, and unusually large meals during the preceding 24 h.

#### 2.7.3. Blood Sampling and Biochemical Analysis

Capillary blood samples were collected by finger prick at baseline (0 min) and at 30, 60, and 120 min following consumption of the test meal. Blood glucose concentrations were measured using an Accu-Chek Performa glucometer (Roche Diagnostics, Mannheim, Germany), based on the glucose oxidase method. Quality control was performed on each study day using manufacturer-provided control solutions in accordance with ISO 15197 recommendations [17]. The first drop of blood was discarded before sample collection, and all measurements were performed by trained personnel according to the manufacturer’s instructions.

Venous blood samples were collected at the same time points for serum insulin determination. Samples were centrifuged immediately after collection, and the separated serum aliquots were stored at −70 °C until analysis. Serum insulin concentrations were measured in duplicate using the Invitrogen™ Human Insulin ELISA Kit (Catalogue No. KAQ1251; Life Technologies Corporation, Frederick, MD, USA) according to the manufacturer’s instructions. The assay had an analytical sensitivity of 0.17 µIU/mL, with intra-assay and inter-assay coefficients of variation of 5.4% and 8.5%, respectively.

To minimize inter-assay variability, all serum samples were analyzed within a single analytical run by the same experienced operator.

#### 2.7.4. Calculation of Postprandial Responses

Postprandial glycemic and insulinemic responses were assessed by calculating the incremental area under the curve (iAUC) using the trapezoidal method, considering only values above baseline.

Because the control wheat chapatti, rather than a glucose reference solution, was used as the comparator, relative glycemic and insulinemic responses were calculated instead of standardized glycemic and insulinemic indices.

The Relative Glycemic Response (RGR) was calculated as:

RGR (%) = (iAUCtest meal/iAUCcontrol chapatti) × 100

The Relative Insulinemic Response (RIR) was calculated as:

RIR (%) = (iAUCtest meal/iAUCcontrol chapatti) × 100

These indices were used to compare the metabolic effects of sprouted and unsprouted fenugreek supplementation relative to the control chapatti under identical experimental conditions.

### 2.8. Statistical Analysis

All laboratory analyses were performed in triplicate. Data are presented as mean ± standard deviation (SD). Differences among treatments were evaluated using one-way analysis of variance (ANOVA) followed by Fisher’s least significant difference (LSD) post hoc test. Colour measurements were analyzed using one-way analysis of variance (ANOVA), followed by Fisher’s least significant difference (LSD) post hoc test to compare differences among formulations. Repeated-measures ANOVA was used to assess changes in glycemic and insulinemic responses over time. Pearson’s correlation analysis was performed to evaluate associations between variables. Statistical significance was established at *p* < 0.05. All analyses were conducted using GraphPad Prism version 8.0 (GraphPad Software, San Diego, CA, USA). The crossover design allowed each participant to act as his or her own control, thereby minimizing between-subject variability. Potential period and carry-over effects were reduced through randomization of treatment order and the inclusion of a washout period between interventions. As this was a pilot study, no a priori sample size calculation was performed. Therefore, the findings should be interpreted with caution and confirmed in larger, adequately powered studies.

## 3. Results

### 3.1. Effects of Sprouting on Nutritional Composition

Sprouting induced significant changes in the proximate composition of both wheat grains and fenugreek seeds.

**Figure 1:**
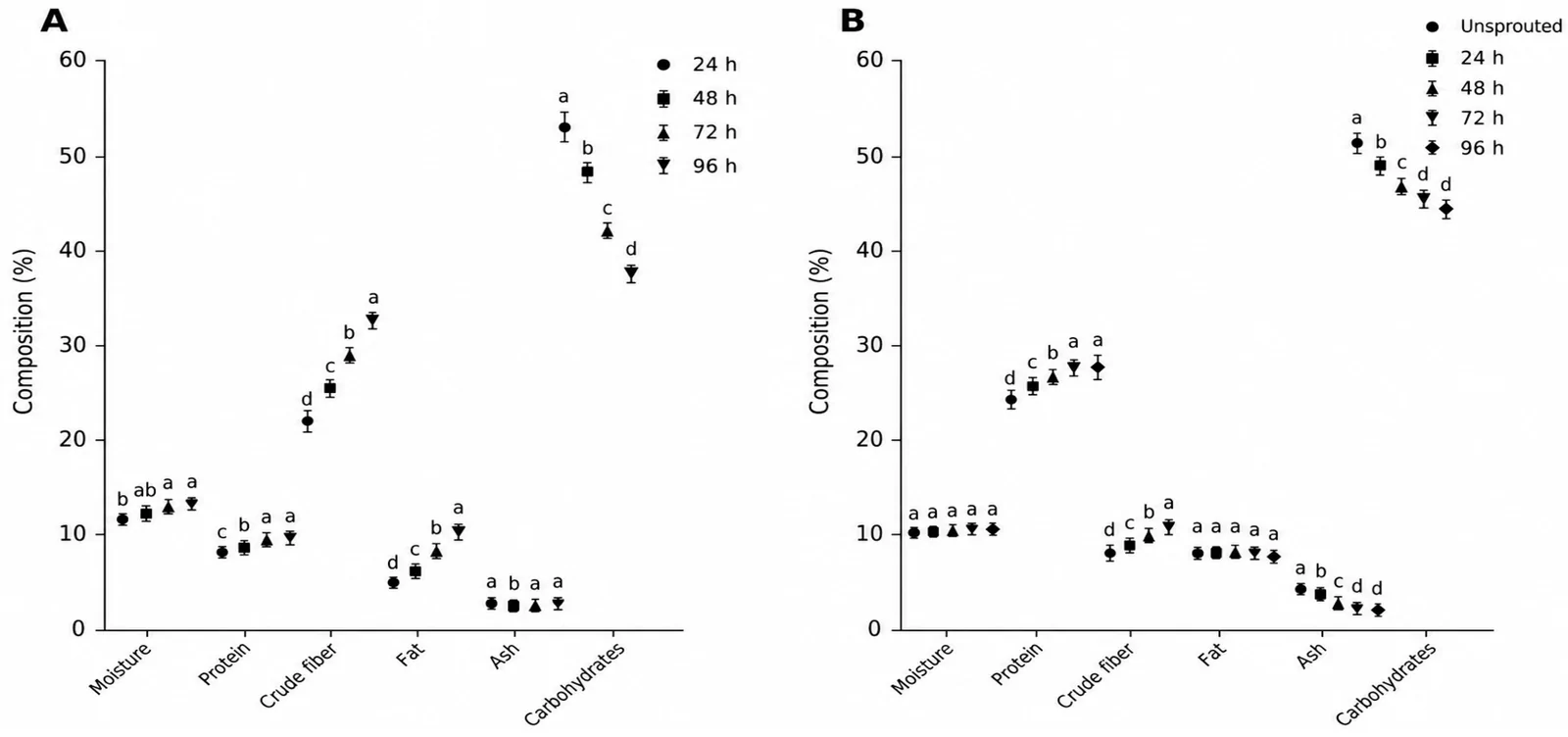
Effect of sprouting on the nutritional composition (% dry weight) of (**A**) wheat grains at different germination times (24–96 h) and (**B**) fenugreek seeds (unsprouted vs. sprouted). Data are presented as mean ± SD (*n* = 3). Different lowercase letters indicate statistically significant differences among sprouting treatments within each nutritional parameter, as determined by one-way ANOVA followed by Fisher’s least significant difference (LSD) post hoc test (*p* < 0.05).

In wheat grains, increasing sprouting duration (24–96 h) resulted in statistically significant modifications in several compositional parameters (Figure 1A). Moisture content increased from 11.2 ± 0.48% at 24 h to 12.83 ± 0.14% at 96 h (*p* < 0.05). Protein content also showed a significant increase (*p* < 0.001), rising from 7.48 ± 0.08% to 8.84 ± 0.29%. Similarly, crude fiber increased markedly from 22.38 ± 0.03% to 31.43 ± 0.01% (*p* < 0.001), while fat content rose from 3.98 ± 0.01% to 7.70 ± 0.08% (*p* < 0.001). In contrast, ash content did not change significantly (*p* > 0.05), whereas carbohydrate content decreased significantly with increasing sprouting time. These results reflect the metabolic and biochemical transformations occurring during germination.

For fenugreek seeds, comparison between unsprouted and sprouted samples revealed significant increases in protein and crude fiber content (Figure 1B). Protein increased from 22.75 ± 0.13% to 24.98 ± 0.20% (*p* < 0.001), while crude fiber rose from 7.68 ± 0.03% to 9.17 ± 0.16% (*p* < 0.001). Conversely, carbohydrate content decreased following sprouting. No significant differences were observed in moisture or fat content between unsprouted and sprouted fenugreek seeds.

### 3.2. Colour Profiling of Wheat and Sprouted and Un-Sprouted Fenugreek Supplemented Chapattis

Color attributes were characterized using CIE Lab* parameters. Increasing sprouted fenugreek levels decreased lightness (L*) from 75.02 (control) to 68.10 (sprouted 7.5%) and increased redness (a*) from 7.49 to 9.68. Yellow–blue (b*) value also increased slightly (Table 1). Un-sprouted fenugreek at similar levels produced higher L* and b* values, indicating lighter, more yellow products, but with increased bitterness. Color changes reflected the incorporation of fenugreek and influenced panelist preference.

### 3.3. Antioxidant Properties of Sprouted and Unsprouted Samples

**Figure 2:**
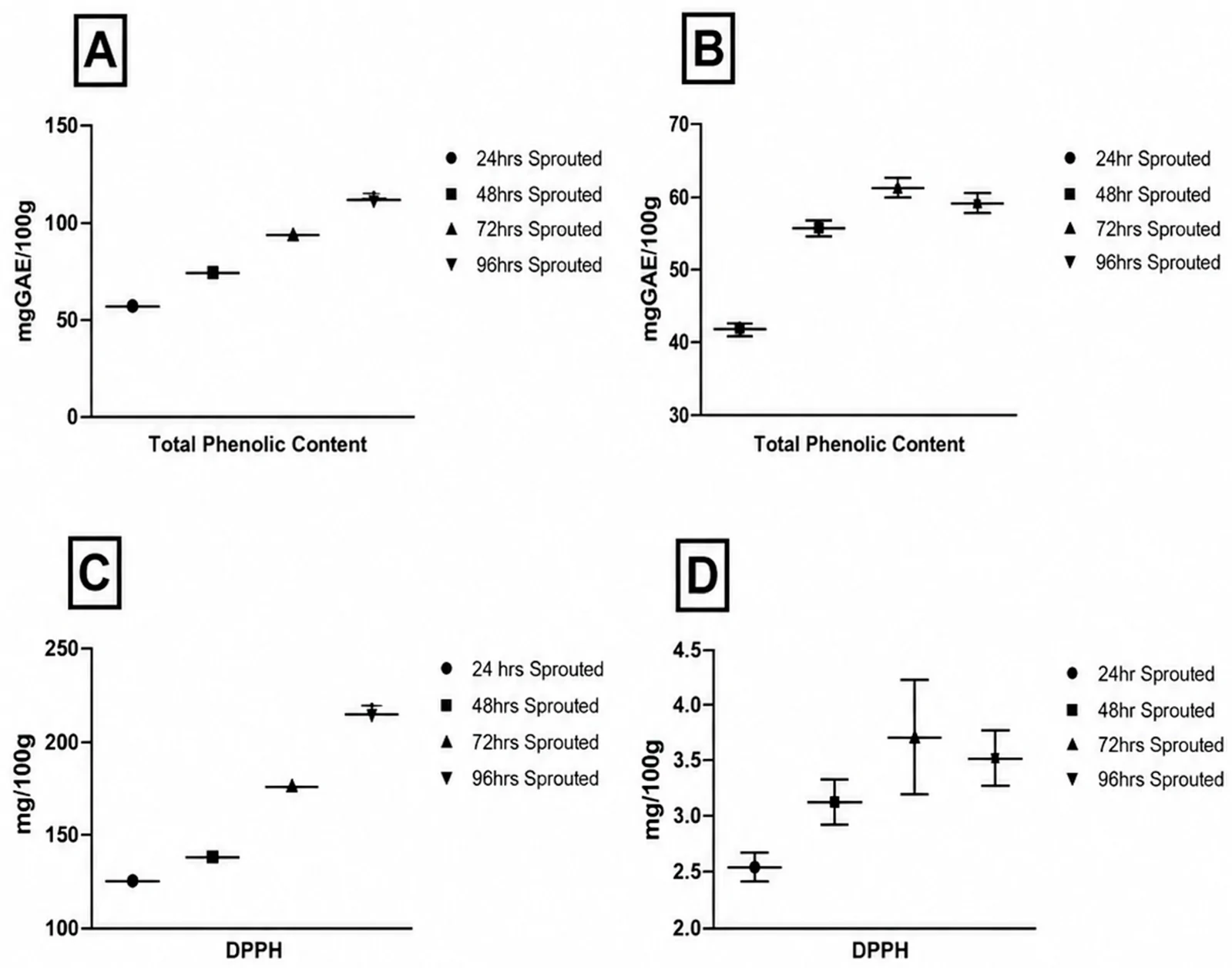
Effect of sprouting on antioxidant properties. (**A**) Wheat TPC; (**B**) fenugreek TPC; (**C**) wheat DPPH; (**D**) fenugreek DPPH. Sprouting significantly increased total phenolic content and radical scavenging activity compared to unsprouted samples. Data are presented as mean ± standard deviation (*p* < 0.001).

Sprouting significantly enhanced the antioxidant properties of both wheat and fenugreek, as evaluated by total phenolic content (TPC) and DPPH radical scavenging activity. In wheat, antioxidant activity increased markedly with sprouting duration (Figure 2A). TPC nearly doubled, rising from 55.82 ± 0.22 to 110.17 ± 0.52 mg GAE/100 g between 24 and 96 h (*p* < 0.001). Similarly, in (Figure 2C) DPPH radical scavenging activity increased from 125.3 ± 0.5 to 209.56 ± 0.5 mg/100 g over the same period (*p* < 0.001). These findings are consistent with previous studies showing that germination enhances antioxidant capacity in wheat. Calzuola et al. reported that antioxidant activity increases progressively during germination, with peak levels observed after 3–5 days [14].

In Fenugreek, (Figure 2B) sprouting for 96 h significantly increased antioxidant potential. TPC rose from 30.0 ± 0.5 to 61.09 ± 0.95 mg GAE/100 g (*p* < 0.001), while (Figure 2D) DPPH activity increased from 2.04 ± 0.09 to 3.61 ± 0.43 mg TE/100 g. These results confirm the positive impact of sprouting on antioxidant capacity.

The observed increase in antioxidant activity may be attributed to changes in phenolic compounds during germination. Previous studies have shown that free phenolic acids increase, while bound phenolics may initially decrease during soaking and subsequently rise during germination. In this context, DPPH activity is widely used as an indicator of antioxidant capacity in food systems [15].

### 3.4. Demographic and Clinical Features of Subjects

The clinical and demographic characteristics of the study participants are presented in Table 2. A total of 18 healthy, normoglycemic young adults (9 males and 9 females) were included in the study. The mean age was 23.5 ± 2.07 years, and the mean body mass index (BMI) was 21.9 ± 1.7 kg/m^2^. The average fasting blood glucose concentration was 82.8 ± 4.7 mg/dL, confirming normal glycemic status. All participants underwent anthropometric assessment and clinical screening prior to enrollment and provided written informed consent.

### 3.5. Organoleptic Properties and Sensory Evaluation

Bitterness associated with fenugreek and sprouting was a key factor influencing panel acceptance. Similar observations have been reported in previous studies.

Sensory evaluation revealed significant differences among formulations (Table 3). Chapattis containing 7.5% sprouted fenugreek (T_3_) achieved the highest overall acceptability among the experimental samples (7.52 ± 0.12), with favorable scores for taste, texture, and folding ability. Control chapattis (T_0_) received the highest overall score (8.00 ± 0.47), while lower inclusion levels (T_1_ and T_2_) showed reduced but still acceptable ratings.

Formulations exceeding 7.5% resulted in increased bitterness and reduced acceptability, indicating that 7.5% represents the optimal inclusion level balancing sensory quality and functional benefits.

### 3.6. Glycemic Response in Healthy Subjects

**Figure 3:**
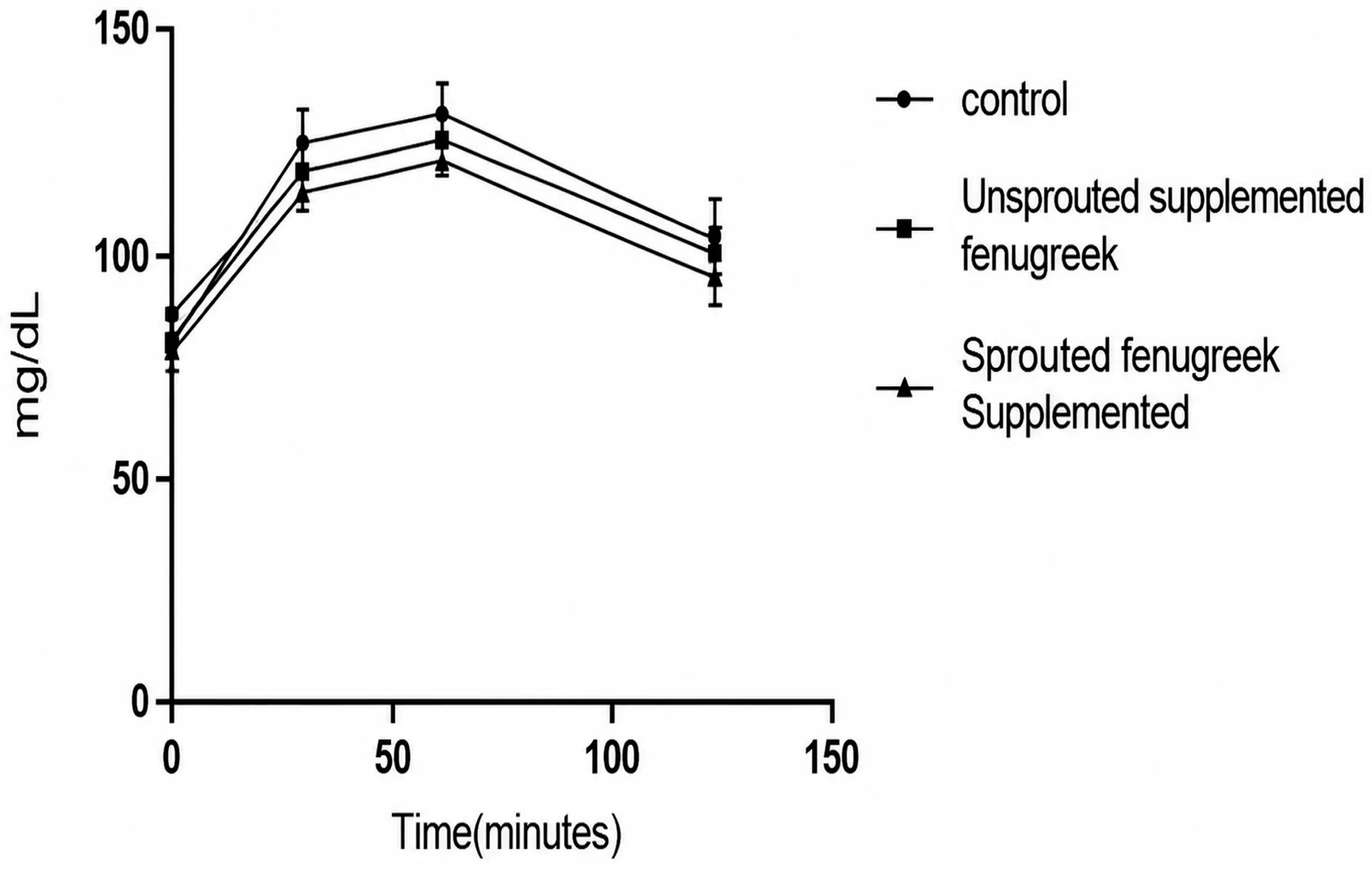
Postprandial glycemic response following consumption of control, sprouted, and un-sprouted fenugreek chapattis. Blood glucose levels were measured at 0, 30, 60, and 120 min. Sprouted fenugreek significantly reduced peak glucose levels and overall glycemic response compared to control. Values are expressed as mean ± standard deviation (*p* < 0.05).

Eighteen healthy volunteers completed the postprandial blood glucose evaluation. The glycemic response curves for control, sprouted fenugreek, and unsprouted fenugreek chapattis are shown in Figure 3.

Consumption of the control wheat chapatti resulted in a rapid increase in blood glucose concentration, reaching a peak value of approximately 120 mg/dL at 30 min, followed by a gradual decline toward baseline by 120 min. In contrast, the sprouted fenugreek chapatti (7.5%) produced a significantly lower peak glucose concentration (95 mg/dL; *p* < 0.05 versus control) and a reduced overall postprandial glycemic excursion. The unsprouted fenugreek chapatti also reduced peak glucose concentration (105 mg/dL), although to a lesser extent than the sprouted formulation.

The incremental area under the glucose response curve (iAUC) was reduced by 36.6% in participants consuming the sprouted fenugreek chapatti compared with the control chapatti (*p* < 0.001), whereas the unsprouted fenugreek chapatti reduced iAUC by 20.5%.

The absolute iAUC values and 95% confidence intervals for all three test conditions are summarized in Table 4. The sprouted fenugreek chapatti produced the lowest glucose iAUC (1810.7 ± 271.6 mg/dL·min; 95% CI: 1675.6–1945.8), corresponding to a Relative Glycemic Response (RGR) of 63.4% relative to the control chapatti, while the unsprouted formulation achieved an intermediate response (2270.5 ± 340.6 mg/dL·min; 95% CI: 2101.1–2439.9; RGR = 79.5%).

Repeated-measures ANOVA revealed significant effects of treatment and time on postprandial blood glucose concentrations (*p* = 0.008 and *p* = 0.0001, respectively), whereas the treatment × time interaction was not significant (*p* = 0.18).

These findings suggest that supplementation with fenugreek, particularly in its sprouted form, may improve postprandial glucose handling and contribute to better metabolic control. These results are consistent with previous reports demonstrating that fenugreek attenuates postprandial glycemic responses through mechanisms including delayed carbohydrate digestion, reduced intestinal glucose absorption, and modulation of glucose-metabolizing pathways [16].

### 3.7. Insulinemic Response

Postprandial serum insulin patterns closely mirrored the glycemic responses. Consumption of the control wheat chapatti produced a peak insulin concentration of 45 μIU/mL at 30 min, whereas the chapatti supplemented with sprouted fenugreek reached only 32 μIU/mL (*p* < 0.01 vs. control), and the chapatti supplemented with unsprouted fenugreek reached 38 μIU/mL. Both fenugreek formulations attenuated postprandial insulin excursions and accelerated the return to baseline values.

**Figure 4:**
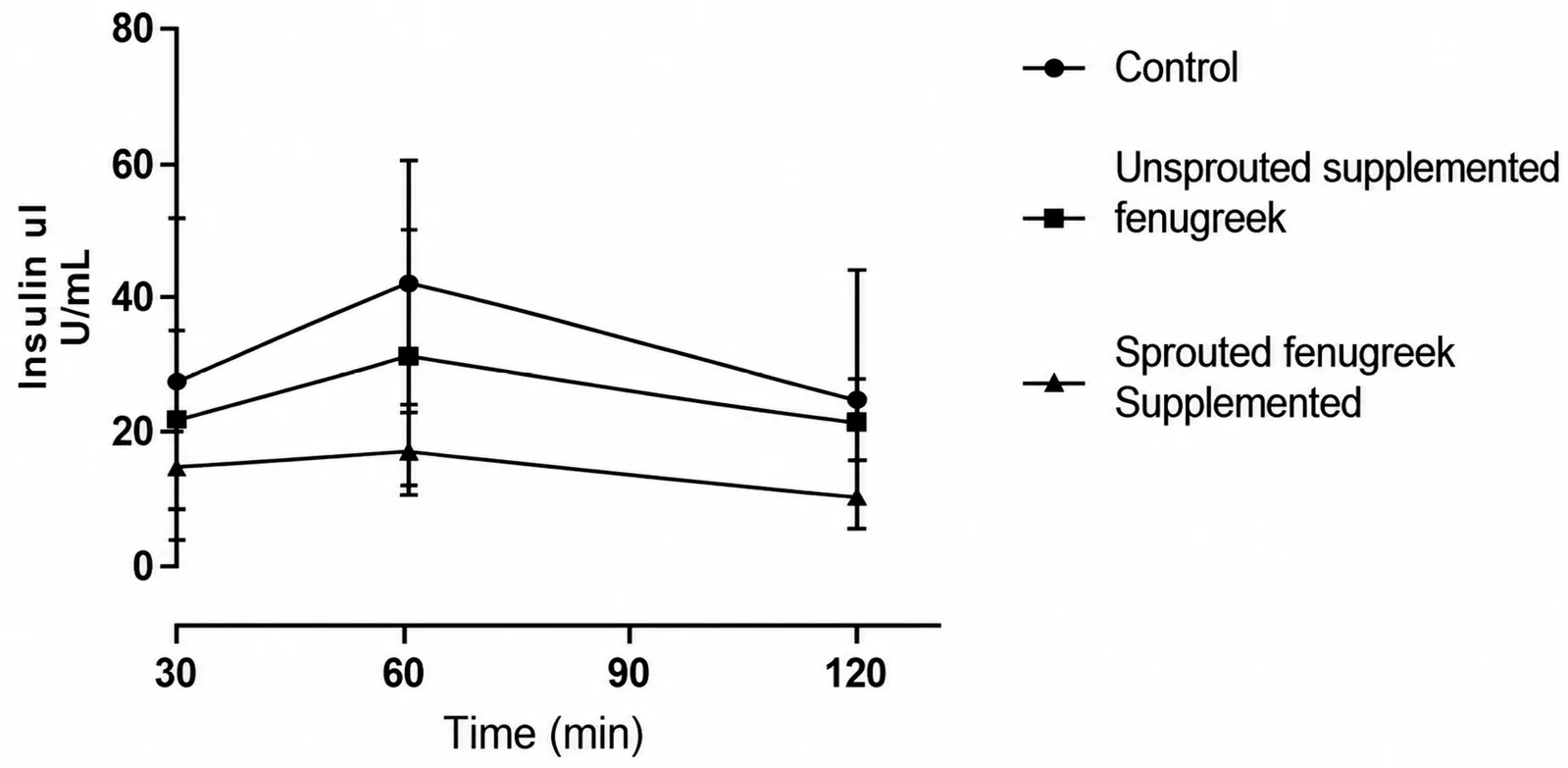
Postprandial serum insulin response following consumption of control, sprouted fenugreek, and unsprouted fenugreek chapattis. Sprouted fenugreek significantly reduced serum insulin concentrations at 30, 60, and 120 min compared with the control chapatti. Values are expressed as mean ± SD.

Figure 4 summarizes the postprandial insulin responses for all treatments. Sprouted fenugreek consistently reduced serum insulin concentrations at 30, 60, and 120 min compared with the control chapatti, whereas unsprouted fenugreek produced a more modest reduction at the early postprandial time points and approached control values by 120 min. Overall, insulin concentrations at 30 and 60 min were lower for both fenugreek-supplemented chapattis, but only the sprouted fenugreek formulation maintained a pronounced reduction at 120 min. Statistical analysis revealed significant effects of treatment and time (*p* = 0.002–0.007), whereas the treatment × time interaction was not significant (*p* = 0.4).

As shown in Table 4, the Relative Insulinemic Response (RIR) was reduced to 65.7% for the sprouted fenugreek chapatti and to 77.9% for the unsprouted fenugreek chapatti, corresponding to reductions of 34.3% and 22.1%, respectively, compared with the control chapatti. These findings indicate that sprouted fenugreek is particularly effective in attenuating postprandial insulinemic responses and reducing insulin demand following carbohydrate-rich meals. The parallel reductions in postprandial glucose and insulin responses suggest an improvement in metabolic efficiency, supporting the potential use of sprouted fenugreek as a functional ingredient for the development of foods aimed at promoting glycemic control and metabolic health.

## 4. Discussion

The present study demonstrates that the incorporation of sprouted fenugreek into wheat-based flatbread significantly improves the nutritional profile of the product and attenuates acute postprandial glycemic and insulinemic responses in healthy adults. These findings support the potential use of sprouted fenugreek as a functional ingredient for the development of cereal-based staple foods with improved metabolic properties.

Sprouting markedly enhanced the nutritional quality of both wheat and fenugreek, as shown by increased protein, dietary fiber, total phenolic content, and antioxidant activity. These changes are consistent with previous evidence indicating that germination activates endogenous hydrolytic enzymes, promotes the degradation of complex macromolecules, and increases the release or biosynthesis of bioactive compounds, including phenolic acids and antioxidant molecules [18]. In addition, sprouting is known to reduce antinutritional factors, particularly phytates, thereby improving mineral bioaccessibility and the nutritional value of cereal- and legume-based products [19]. The reduction in postprandial glucose response observed after consumption of the sprouted fenugreek-enriched chapatti is consistent with previous human studies showing that fenugreek can reduce postprandial glycemia when incorporated into carbohydrate-rich foods. In particular, the replacement of refined wheat flour with fenugreek seed powder has been reported to significantly reduce the glycemic response of buns and flatbreads [20]. These findings are also supported by broader clinical evidence suggesting beneficial effects of fenugreek intake on glycemic control, especially in individuals with impaired glucose metabolism or type 2 diabetes.

Although previous studies have demonstrated that the incorporation of fenugreek seed powder into cereal-based foods can attenuate postprandial glycemic responses, the present study differs in several important aspects. Unlike earlier investigations, we evaluated sprouted fenugreek, a biologically modified ingredient obtained through germination, which markedly enhanced the phenolic content and antioxidant capacity while simultaneously improving the sensory acceptability of the final product. To our knowledge, this is one of the first randomized crossover human studies demonstrating that sprouted fenugreek is more effective than unsprouted fenugreek in reducing both postprandial glucose and insulin excursions when incorporated into a commonly consumed wheat-based flatbread. These findings suggest that germination represents an effective strategy for enhancing the functional properties of fenugreek while maintaining consumer acceptability.

The mechanisms underlying these effects are likely multifactorial. Fenugreek seeds are rich in soluble dietary fiber, particularly galactomannan, which increases intestinal viscosity, delays gastric emptying, and slows carbohydrate digestion and glucose absorption. This may explain the lower postprandial glucose peak and reduced glucose iAUC observed in the present study. Moreover, fenugreek contains bioactive compounds such as 4-hydroxyisoleucine, saponins, and polyphenols, which have been associated with improved insulin sensitivity, modulation of glucose metabolism, and antioxidant effects. Therefore, the metabolic improvement observed after consumption of the sprouted fenugreek chapatti may result from the combined effects of delayed carbohydrate digestion, reduced intestinal glucose absorption, and improved peripheral glucose handling.

The enhanced antioxidant properties observed after germination may also have contributed to the more favorable postprandial metabolic response. Sprouting significantly increased the total phenolic content and radical-scavenging activity of both wheat and fenugreek, suggesting greater availability of antioxidant bioactive compounds in the enriched flatbread. Oxidative stress can impair insulin receptor signaling through the activation of stress-sensitive pathways and the inhibition of key components of the insulin signaling cascade, thereby reducing peripheral glucose uptake. Phenolic compounds may counteract these effects by limiting reactive oxygen species formation, preserving insulin receptor substrate and PI3K/AKT signaling, and supporting glucose transporter translocation in insulin-responsive tissues. In addition, some polyphenols may attenuate postprandial glycemia by inhibiting carbohydrate-digesting enzymes, including α-amylase and α-glucosidase, and by slowing intestinal glucose absorption. Therefore, the lower glucose and insulin excursions observed after consumption of the sprouted fenugreek-enriched chapatti may reflect the combined action of soluble dietary fiber and germination-induced increases in phenolic compounds and antioxidant capacity. However, because circulating oxidative stress biomarkers and insulin signaling pathways were not directly assessed in the present study, this mechanistic interpretation should be considered plausible but not conclusive. These findings are consistent with recent reviews highlighting that the enrichment of foods with fenugreek-derived bioactive compounds may enhance their functional properties and contribute to improved glycemic regulation through complementary antioxidant and metabolic mechanisms [21].

An important finding of the present study is that sprouted fenugreek exerted a stronger effect than unsprouted fenugreek on both glycemic and insulinemic responses. This suggests that germination may potentiate the metabolic effects of fenugreek by improving the bioavailability of its bioactive compounds and reducing antinutritional factors. Germination may also modify the food matrix, increasing fiber functionality and antioxidant potential, which could contribute to a slower digestion rate and improved postprandial metabolic control. However, the precise contribution of each component remains to be clarified.

The reduction in postprandial insulin response is also relevant. The lower insulin excursions observed after consumption of the sprouted fenugreek-enriched chapatti suggest a reduced insulin demand for maintaining glucose homeostasis. This finding may be particularly important in the context of insulin resistance prevention, as repeated postprandial hyperinsulinemia is considered a metabolic stressor that may contribute to β-cell dysfunction over time. The parallel reduction in glucose and insulin responses observed in this study indicates that the enriched chapatti may improve postprandial metabolic efficiency rather than simply stimulating greater insulin secretion.

From a food technology and public health perspective, the use of chapatti as a delivery matrix is particularly relevant. Wheat-based flatbreads are widely consumed in South Asian diets and represent a major source of daily carbohydrate intake. Reformulating such staple foods with metabolically active ingredients may therefore provide a practical and culturally acceptable strategy for improving dietary quality. Importantly, the 7.5% sprouted fenugreek formulation showed good sensory acceptability, indicating that the functional benefits were achieved without markedly compromising palatability. This is a critical point, as excessive fenugreek incorporation may increase bitterness and reduce consumer acceptance.

Nevertheless, the present study has some limitations. First, the sample size was limited, and the study population included only young, healthy, normoglycemic adults. Therefore, the findings should be considered exploratory and may not be directly generalizable to older individuals or patients with impaired glucose tolerance or type 2 diabetes. Second, only acute postprandial responses were evaluated; thus, the long-term metabolic effects of regular consumption remain unknown. Third, although the study suggests a beneficial role of sprouted fenugreek, the molecular mechanisms responsible for the observed effects were not directly investigated.

Future studies should include larger and more diverse cohorts, including individuals with insulin resistance, prediabetes, or type 2 diabetes. Long-term intervention trials are also needed to determine whether regular consumption of sprouted fenugreek-enriched flatbread can improve fasting glucose, HbA1c, insulin sensitivity, lipid profile, oxidative stress, and inflammatory markers. In addition, further mechanistic studies should investigate starch digestibility, gastric emptying, gut hormone responses, and the bioavailability of fenugreek-derived bioactive compounds. Overall, the present findings provide further evidence that sprouted fenugreek may represent a valuable functional ingredient for improving the nutritional and metabolic properties of cereal-based foods, warranting further investigation in larger and longer-term clinical studies.

## 5. Conclusions

This study demonstrates that fortification of wheat-based chapattis with 7.5% sprouted fenugreek flour significantly enhances nutritional quality, antioxidant capacity, and sensory acceptability, while effectively attenuating postprandial glycemic and insulinemic responses in healthy adults. Germination of both wheat and Fenugreek resulted in increased protein, dietary fiber, and phenolic content, alongside reductions in antinutritional factors, thereby improving the overall functional profile of the final product.

Consumption of sprouted fenugreek-enriched chapattis led to lower postprandial glucose and insulin excursions, with a glucose iAUC reduction of 36.6% and a corresponding Relative Glycemic Response (RGR) of 63.4% compared with the control chapatti. These findings indicate improved metabolic handling of carbohydrates and reduced insulin demand. Importantly, sensory evaluation confirmed that this level of supplementation preserves product acceptability, balancing health benefits with consumer preferences.

Overall, the incorporation of sprouted fenugreek into a widely consumed staple food represents a practical, cost-effective, and culturally adaptable strategy for improving dietary quality and supporting metabolic health. Such functional food approaches may contribute to the prevention and management of type 2 diabetes, particularly in populations with high reliance on cereal-based diets.

Future studies should investigate the long-term metabolic effects of regular consumption in larger and more diverse populations, including individuals with impaired glucose tolerance or type 2 diabetes, to confirm these findings and facilitate their translation into evidence-based dietary recommendations and public health strategies.

## Figures and Tables

**Table 1:** Color profiling of sprouted and unsprouted fenugreek-supplemented chapattis.

Fenugreek Supplementation	L* (Lightness)	a* (Red–Green)	b* (Yellow–Blue)
Control	75.02 ± 2.55 ^ab^	7.49 ± 0.39 ^cd^	28.33 ± 0.25 ^c^
Sprouted 2.5 g	75.34 ± 0.21 ^ab^	7.90 ± 0.05 ^cd^	29.86 ± 1.28 ^bc^
Un-sprouted 2.5 g	77.10 ± 3.25 ^a^	8.27 ± 0.76 ^bc^	34.06 ± 2.13 ^a^
Sprouted 5 g	72.05 ± 0.39 ^bc^	9.02 ± 0.42 ^ab^	31.37 ± 0.76 ^abc^
Un-sprouted 5 g	77.05 ± 0.77 ^a^	7.06 ± 0.51 ^d^	31.98 ± 0.01 ^ab^
Sprouted 7.5 g	68.10 ± 1.57 ^c^	9.68 ± 0.36 ^a^	29.96 ± 1.13 ^bc^
Un-sprouted 7.5 g	69.07 ± 3.33 ^c^	9.06 ± 0.17 ^ab^	33.22 ± 0.02 ^a^

F ratio: L*: 6.45; a*: 4.69; b*: 9.37. Means ± standard deviation. Values with different superscript letters in a column are significantly different at *p* < 0.05.

**Table 2:** Clinical characteristics of study participants.

Clinical Characteristic	Mean ± SD	Range
Number of subjects	18	M:F 9:9
Age (years)	23.5 ± 2.07	20–25
Weight (kg)	62.1 ± 7.78	50–72
Height (cm)	166.1 ± 6.6	160–175
Waist circumference (cm)	78.2 ± 6.4	68–90
BMI (kg/m^2^)	21.9 ± 1.7	20–25
Fasting blood glucose (mg/dL)	82.8 ± 4.7	75–90

Values are mean ± SD or count, with ranges provided where applicable. All participants were normoglycemic, aged 18–25, and medically screened prior to inclusion.

**Table 3:** Sensory evaluation of wheat–fenugreek sprouted composite flour chapattis (9-point hedonic scale).

Sample	Taste	Color	Texture	Folding Ability	Odor	Chewability	Overall Acceptability
T_0_ (0%)	8.0 ± 0.96 ^a^	8.0 ± 1.02 ^a^	8.0 ± 0.77 ^a^	8.0 ± 0.69 ^a^	8.0 ± 0.79 ^a^	8.0 ± 1.02 ^a^	8.0 ± 0.47 ^a^
T_1_ (2.5%)	7.14 ± 0.96 ^c^	6.57 ± 1.02 ^c^	7.01 ± 0.77 ^c^	7.14 ± 0.69 ^c^	7.03 ± 0.79 ^c^	7.27 ± 1.02 ^c^	7.03 ± 0.47 ^c^
T_2_ (5%)	6.54 ± 0.85 ^d^	6.64 ± 0.81 ^c^	6.35 ± 0.80 ^d^	6.85 ± 0.84 ^d^	6.32 ± 0.86 ^d^	6.66 ± 0.85 ^d^	6.56 ± 0.54 ^d^
T_3_ (7.5%)	7.55 ± 0.64 ^b^	7.37 ± 0.5 ^b^	7.40 ± 0.40 ^b^	7.65 ± 0.43 ^b^	7.76 ± 0.34 ^b^	7.43 ± 0.39 ^b^	7.52 ± 0.12 ^b^

Values are mean ± SD (*n* = 24). Point hedonic scale: 9 = like extremely, 1 = dislike extremely. The percentages refer to the concentrations of sprouted. Values with different superscript letters in a column are significantly different at *p* < 0.05.

**Table 4:** Relative glycemic and insulinemic responses following consumption of fenugreek-enriched chapattis.

Treatment	Relative Glycemic Response (RGR, %)	Reduction vs. Control (%)	Relative Insulinemic Response (RIR, %)	Reduction vs. Control (%)
Control chapatti (0% fenugreek)	100.0	—	100.0	—
7.5% sprouted fenugreek chapatti	63.4	36.6	65.7	34.3
7.5% unsprouted fenugreek chapatti	79.5	20.5	77.9	22.1

Relative Glycemic Response (RGR) and Relative Insulinemic Response (RIR) were calculated using the control chapatti as the reference food (100%). Values were derived from the incremental area under the curve (iAUC) calculated using the trapezoidal method, considering only postprandial values above baseline.

## Data Availability

The original contributions presented in this study are included in the article. Further inquiries can be directed to the corresponding authors.
